# Pectin and Pectin-Based Composite Materials: Beyond Food Texture

**DOI:** 10.3390/molecules23040942

**Published:** 2018-04-18

**Authors:** Claudia Lara-Espinoza, Elizabeth Carvajal-Millán, René Balandrán-Quintana, Yolanda López-Franco, Agustín Rascón-Chu

**Affiliations:** Research Center for Food and Development, CIAD, A.C., Carretera a La Victoria Km. 0.6, Hermosillo, Sonora 83304, Mexico; claudialaraes@gmail.com (C.L.-E); ecarvajal@ciad.mx (E.C.-M.); rbalandran@ciad.mx (R.B.-Q.); lopezf@ciad.mx (Y.L.-F.)

**Keywords:** pectin, pectin composites, therapeutic properties, drug delivery system

## Abstract

Pectins are plant cell wall natural heteropolysaccharides composed mainly of α-1-4 d-galacturonic acid units, which may or may not be methyl esterified, possesses neutral sugars branching that harbor functional moieties. Physicochemical features as pH, temperature, ions concentration, and cosolute presence, affect directly the extraction yield and gelling capacity of pectins. The chemical and structural features of this polysaccharide enables its interaction with a wide range of molecules, a property that scientists profit from to form new composite matrices for target/controlled delivery of therapeutic molecules, genes or cells. Considered a prebiotic dietary fiber, pectins meetmany regulations easily, regarding health applications within the pharmaceutical industry as a raw material and as an agent for the prevention of cancer. Thus, this review lists many emergent pectin-based composite materials which will probably palliate the impact of obesity, diabetes and heart disease, aid to forestall actual epidemics, expand the ken of food additives and food products design.

## 1. Introduction

Pectins are plant cell wall structural polysaccharides composed mainly of galacturonic acid units with variations in composition, structure and molecular weight. This polysaccharide is often associated with other cell wall components such as cellulose, hemicellulose and lignin [1]. In general, pectins are located in the primary cell wall and middle lamella of many plants, being in the latter, the one with highest concentration, with a gradual decrease from the primary cell wall towards the plasma membrane [2,3,4].

They are commonly produced during the initial stages of primary cell wall growth and make up about one third of the cell wall dry substances of dicotyledonous and some monocotyledonous plants [5]. Pectins contribute to the firmness and structure of plant tissue, being involved in the intercellular adhesion and mechanical resistance of the cell wall, and they also have an important role in the development of plant cells [6,7], providing turgidity and resistance to low temperatures and drought [8]. These structural polysaccharides have also other important functions in the interactions between plants and pathogens [9]; the amount and nature of pectin are critical for texture in fruits and vegetables during their growth, maturation, storage and processing [7]. They also seem to play a role in controlling plant fluid movement through rapidly growing parts [3].

This versatile polysaccharide has been used in the food and beverage industries for many years. The principal applications of pectin are as a gelling agent, thickening agent, stabilizer and emulsifier [8,10]; pectin also provides an important source of dietary fiber, which may have therapeutic properties [11,12,13]. Besides its importance in the food industry, pectin has potential uses in many other fields, like medicine, as a carrier for controlled drugs or bioactive release [8,14], for instance, in nasal, ocular and oral drug delivery [15,16,17] and wound healing [18].

Diverse biopolymers of great importance for the pharmaceutical industry include proteins, chitosan, starch, and gelatin. Gelatin has the capacity to form hydrogels for water purification [19], while starch blends with minerals have some biomedical applications as bone substitution materials [20]. Chitosan is also widely used in biomedical and pharmaceutics as tissue engineering and wound healing [21]. In the present review, the capacity of pectins to interact with such beneficial molecules results in new composite materials with immediate applications. The functional groups present in the pectin structure that allow the interaction with a variety of molecules are briefly presented in the sections below.

### 1.1. Chemical Structure

The exact chemical composition and structure of pectin is still under debate due to the high complexity of this molecule. Elucidation of pectin structure is important to understand its role in plant growth and development, during ripening of fruits, in food processing, and as a nutritional fiber. The structure of pectins is very difficult to determine because their composition varies with the source and conditions of extraction, location, and other environmental factors. Pectin can also change during its isolation from plants, storage, maturity degree, and processing of raw plant material [22,23]. At present, the consensus states that pectins are heterogeneous polysaccharides with three main structural domains: homogalacturonan, alternating with two types of highly branched rhamnogalacturonans regions designated as RG-I and RG-II [24,25]. Other structural classes of pectic polysaccharides are also included, such as xylogalacturonans, arabinogalactans and arabinans [26,27].

#### 1.1.1. Homogalacturonan

Homogalacturonan consists in linear polymers, composed mainly of d-galacturonic acid units (at least 65%) joined in chain by α-(1-4)-glycosidic linkages [13,28,29]. The carboxyl groups present at C-6 in the galacturonic acid units can be partially methylesterified and the free acid groups may be partly or fully neutralized with sodium, potassium or ammonium ions. Pectins may also be acetylated onthe *O*-2 or *O*-3 positions, depending of the source [30].

According to the amount of carboxyl groups that can be esterified with methyl groups, pectins are classified on the basis of their degree of esterification [31], also known as degree of methoxylation. Pectins with more than 50% of the carboxyl groups esterified are named as high methoxyl (HM) and pectins with less than 50% of carboxyl groups esterified are classified as low methoxyl (LM) [32,33]. This feature is directly related to the gelling mechanism and therapeutic properties [29,34,35]; this classification will be described down the following section.

Xylogalacturonans (XGA) are homolagacturonans substituted with a β-linked d-xylose-(1-3) at *O*-3, which in turn is occasionally substituted at *O*-4 with an additional β-linked d-xylose [36,37]. Mohnen has reported that the proportion of xylopyranosul residues to galactosyluronic acid residues ranges from 40 to 90% [38]. Part of the galacturonan backbone in XGA region is methylesterified independently of the xylose substitutions [39,40]. The degree of xylosidation may vary depending of the fruit, i.e., the xylosidation degree of watermelon can vary between 25% and 75% from apple [40,41,42]. Xylogalacturonans have been mainly identified in reproductive tissues such as fruit and seeds and are associated with functions including storage and reproduction plant organs [36,39].

#### 1.1.2. Rhamnogalacturonan-I (RG-I)

RG-I is a family of pectic polysaccharides that contain a backbone of repeating [→α-d-GalpA-1,2-α-l-Rhap-1,4→]_n_ units [43]. RG-I is abundantly substituted by neutral sugars like arabinose and galactose forming arabinan, galactan and arabinogalactans in the side chains, predominantly attached to the *O*-4 position of rhamnose [13,44]; although their relative proportions and chain lengths may differ depending on the plant source [45,46]. The RG-I backbone may contain up to 300 rhamnosyl and 300 galactosyluronic acid residues [30]. The highlybranched nature of RG-I has led to the name “hairy region”.

Arabinogalactans are found in two structural different forms, named arabinogalactans I (AGI) and arabinogalactans II (AGII). Type I arabinogalactans consist in a linear chain of 1,4-linked β-d-galactose, containing up to 25% α-l-arabinose residues 1,5-linked in short side chains, connected predominantly to *O*-4 of the rhamnosyl residues [30]. Also single galactose substitutions at *O*-6 have been found. The AGI backbone can be terminated with α-l-arabinose-(1→4) at the non-reducing end [47]. This type of arabinogalactan has been isolated from citrus, potatoes, soybean, lupin, apple, onion, tomato, cabbage and kiwi [48].

Arabinogalactans type II is composed of ramified chains of a 1,3-linked β-d-galactose backbone, containing short side chains of α-l-arabinose-(1→6)-[β-d-galactose-(1→6)]_n_ (*n* = 1, 2, or 3) [24,26,49]. The galactosyl residues of the side chains can be substituted with α-l-arabinose-(1→3) residues. As a result, arabinogalactan type II contains chains of β-d-galactose, joined predominantly by 1,3-linkages in the interior chains, and mainly by 1,6-linkages in the exterior chains. In addition, type II arabinogalactans can also be associated with a complex family of proteoglycans (3–8% proteins) known as arabinogalactan proteins (AGPs) [50,51]. The protein part of this molecule is rich in aminoacids like proline/hydroxyproline, serine, and threonine [52].

Arabinans consist of a backbone of 1,5-linked α-l-arabinose residues with α-l-arabinose substitutions attached at the *O*-2 and *O*-3 positions to about one-third of the backbone. They can be unbranched, substituted with single arabinose units, or substituted with short 1,3-linked α-l-arabinose chains [53].

#### 1.1.3. Rhamnogalacturonan-II (RG-II)

RG-II has a highly conserved structure and consistsofa linear backbone chain of galacturonic acid units, substituted with l-rhamnose, d-galactose and many unusual sugars, such as apiose, aceric acid, 3-*O*-methyl-l-fucose, 2-*O*-methyl-d-xylose, 3-*C*-carboxy-5-deoxy-l-xylose, 3-deoxy-d-manno-octulosonic acid and 3-deoxy-d-lyxo-heptulosaric acid [30,46,54]. The side chains of RGII consist of 12 different types of sugars with over 20 different linkages. The structure of RGII, the most structurally complex pectin domain, is largely conserved across many plant species. Cross-linking between RGII chains of two adjacent pectin molecules increases the integrity of the pectin network [38]. Due to the structure, RG-II has the ability to form borate esters dimers [55].

### 1.2. Biosynthesis of Pectin

Though pectins have been reported since the 1800s, pectin biosynthesis is a subject of study still under development due to its complex structure. In fact, pectin synthesis may be regulated in a temporally, spatially and developmentally specific manner since the pectin polysaccharides are largely absent from many secondary walls while they show cell type and developmental expression in primary walls.

**Figure 1:**
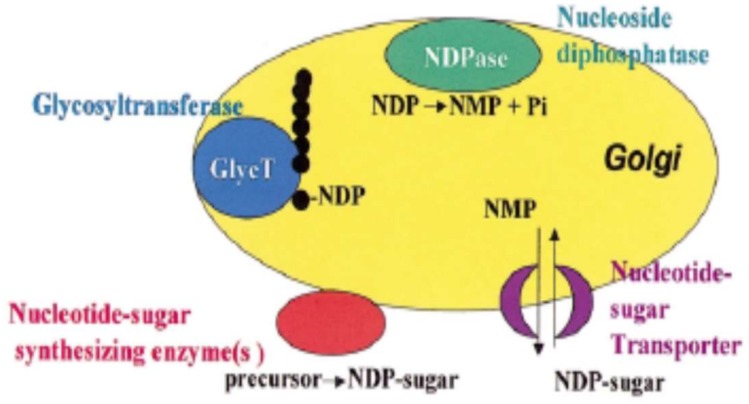
Pectin biosynthesis [26]. The model stablishes that pectin biosynthesis ocurrs in the cytosyolic side of the Golgi apparatus.

Polysaccharides are synthesized in Golgi vesicles, but it cannot be excluded that some initial steps take place in the endoplasmic reticulum or that some assembly steps take place in the cell wall. However, the evidence for Golgi localization is strong, showing that some pectic biosynthetic activities have been shown to cofractionate with Golgi markers; pectic epitopes can also be found in the Golgi vesicles but not in endoplasmic reticulum. Besides, some enzymes identified have been located in the Golgi apparatus [56,57]. Hence, the synthesized pectin is transported to the wall in membrane vesicles (Figure 1).

The Golgi apparatus is a complex organelle formed of vesicles containing proteins and has four defined regions named, *cis-*, medial, *trans*-Golgi and the *trans*-Golgi network. The synthesis occur simultaneously in different Golgi stacks through the *cis-*, medal and *trans*-Golgi cisternae [58]. Glycosyltransferases enzymes transfer glycosyl residues from nucleotide-sugars into polysaccharide acceptors in the pectin synthesis occurring in the Golgi lumen. Some glycosyl residues are modified during the synthesis. In example, the esterification or *O*-methylation is catalized by methyltranferase and acetylation by acetyltransferase enzyme and in some *Chenopodiaceae* (e.g., spinach, sugar beet) feruloylation is catalized by feruloyltranferases [59].

As mentioned above, due to complexity of pectin structures, a large number of enzymes are required for pectin synthesis. Mohnen et al. [60] estimated that around 67 enzymes like glycosyltransferases, methyltransferases, and acetyltranferases are required and multiple activities are necessary for the synthesis of HG, RG-I and RG-II.

Methylesterases act on the highly methylesterified HG present in the cell wall and is deesterified to some degress [61]. This conversion has been associated with the cessation of growth [62], and with the association of HG molecules to each other via calcium binding. These ionic bonds contribute to cell-cell adhesion. Also it has been shown that HG in the middle lamella can be unesterified and in some cell walls some HG regions remains esterified [63]. Different stages of growth define methylesterification patterns. Studies on biochemical and structural data of pectin strongly suggest that HG and RG-II are covalently linked to the cell wall, and HG may act as a backbone for the RG-II synthesis [26]. New approaches will eventually unveil the whole biosynthesis pathway for a well-known polysaccharide.

### 1.3. Sources

#### 1.3.1. Traditional Sources

Pectin can be found in almost all plants, but commercially most pectins are obtained from citrus fruits like orange, lemons, grapefruit, and apples [64,65]. These materials contain high amount of pectic substances and can be found available as residues from juice production. Though, color may be different depending on source of pectin, for technical use is not significant [66].

Fruits like quince, plums, gooseberries, contain much more pectin compared to soft fruits like cherries, grapes and strawberries. Dried apple pulp generally contains 15 to 20% pectin and dried citrus peel range between 30 to 35% of pectin [67]. Another typical levels of pectin in fruit like apricot, cherries, orange and carrots are 1%, 0.4%, 0.5–3.5% and 1.4% respectively, in base of fresh weight [68]. Nevertheless, criteria for commercial production are not only yield, but new properties and applications.

#### 1.3.2. Unconventional Sources

In recent years, the search of new sources of pectin appears very promising. The use of waste by-products obtained from industries has become interesting for extraction of pectin; although without significant commercial use, some examples include sunflower head residues, mango waste, amaranth, olive pomace, and sugar beet pulp [68,69,70,71].

In sugar beet Li et al. [72], reported yields up to 23% of pectin, depending of the extraction conditions. Since then many studies have followed characterization of the polysaccharide. However, pectin from sugar beet has several structural disadvantages as a commercial source of pectin [73]. In spite of its high pectin content, availability, and relatively low cost, sugar beet pectin is little used as a texturizer due to the poor gelling ability compared to apple and citrus pectin. The latter due to high amount of acetyl groups, lower molecular weight, high content of neutral sugars and a higher amount of proteins bound convalently in the lateral chains [74,75,76,77,78]. Although sugar beet pectins cannot form strong gels, they possess excellent emulsifying properties, being superior to pectin extracted from conventional sources [79]. It has been reported that the high protein content influence the emulsifying ability of beet pectins for its capacity to activate the oil-water interface adsorbing favorably on to the surface of oil droplets [80,81].

Also, in contrast to apple and citrus pectin, beet pectin contains ferulic acid residues that allow it to cross-link covalently in presence of an oxidant (chemical or enzymatic) [73,82]. Therefore, sugar beet pectin can be used in applications quite different from those of current commercial pectin, including material that can absorb and hold many times their weight of water [66]. Pectins from olive pomace, as well as beet pectins, have low molecular weight, high content of neutral sugars, and poor gelling activity [83]. This type of pectin is able to gel by ionic interactions with calcium, and shows different gelling properties to those in classic commercial pectins, likely related to the participation of other intermolecular interactions stabilizing the pectin network [83,84,85].

Other potential sources of pectin are sunflower head residues obtained after oil extraction, with very attractive gelling properties for its high molecular weight and high galacturonic acid content [86,87,88]. This type of pectins may contain 3.3 to 5.0% water soluble high-methoxyl pectin and 11.8 to 14.3% insoluble low-methoxyl pectin [89,90]. Other authors also reported a representative quality of pectins in potato pulp [91], a waste material from the potato starch industry pumpkin pulp [92], peach pulp [93,94], (residue from juice industry) and linseed seeds [95] which show attractive yields and properties. Eventually, full characterization of novel pectins will find a way into applications we now only presume to understand.

#### 1.3.3. Ferulated Pectins

Ferulic acid is a component of some plant families, where it can be esterified to cell-wall polysaccharides. The determination of the type and site of linkage between ferulic acid and polysaccharides is based on the analysis of low molecular weight carbohydrate esters of ferulic acid obtained from enzymatic or chemical hydrolysates of cell walls [96].

Numerous studies have been carried out on isolated ferulic esters of hemicellulosic oligosaccharides from *Gramineae* monocots cell walls [97,98,99,100]. Reports on the detailed structure of feruloylated oligosaccharides from dicotyledons are not frequent. Nonetheless *Chenopodiaceae* family shows ferulic acid associated with pectic fractions in cell walls [96,101,102]. The ferulic acid groups are ester-linked with pectins mainly on the *O*-2 position of arabinose residues and, to a lesser extent, on the *O*-6 of galactose residues in side chains of rhamnogalacturonan I [96,103,104]. Ferulic acid is distributed almost equally between the arabinan and galactan components of the pectin side chains [101,105].

Thought sugar beet is the most reported and extensively characterized [70,73,96,101], ferulated pectins have been reported for spinach [70,106,107], quinoa [108], glasswort [109] and amaranth [110]. In sugar beet, the ferulic acid content ranges between 0.8% and 1.0% (*w*/*w*) [101,103,111]; while, Renard et al. [108], found 3.4 mg/g of ferulic acid in quinoa leaves.

The importance of these phenolic compounds lies in its ability to cross-link polysaccharide chains through dimerization reactions, which makes it a very important component for the biology and physiology of the cell wall [102,106,112], mechanical properties in some tissues [113], including extensibility, intercellular adhesion, and cell growth [71,114]. In addition, it has been shown that the amount of ferulic acid released during saponification of cell walls is correlated with microbial [115] and enzymatic [116], degradations of cell-wall polysaccharides, and protects against pathogen invasion [117]. On the second hand, food and non-food industries may find applications for this feature, where linkages are independent from pH and temperature.

### 1.4. Gelling Mechanisms

One of the main characteristics and attractiveness of pectins is their capacity to form gels. The gelation mechanism of pectins is mainly governed by their degree of esterification, so, the mechanism of gel formation is different for both HM and LM pectins [10,29]. The gelling process in pectins is directly affected by both extrinsic and intrinsic factors. These parameters include the degree of methylation, charge distribution along the backbone, average molecular weight, ionic strength, temperature, pH, and the presence of cosolutes [118].

#### 1.4.1. HM Pectins

HM pectins have a degree of esterification typically in a range of 50–80% and require specific conditions to gel, such as low pH (2.5–3.5), and the presence of soluble solids; mainly sucrose (55–75%) or other similar co-solutes (e.g., sorbitol, ethylene glycol) [10,29]. The function of sugar in the formation of gels is to reduce the water activity to stabilize junction zones by promoting hydrophobic interactions. The effect of sugars depends specifically upon the molecular geometry of the sugar and the interactions with neighboring water molecules [119,120].

Such a gel is considered a 2-dimensional network of pectin molecules in which the solvent (water) with the co-solutes sugar and acid are immobilized. This results in a system resisting deformation and showing a stress-strain relationship for small deformation. The buildup of the 3-D network is based on the formation of junction zones stabilized by hydrogen bonding between carboxyl and secondary alcohol groups and by hydrophobic interactions between methyl esters [121]. HM-pectin gels are thermally reversible. In general, HM-pectins are hot water soluble and often contain a dispersion agent such as dextrose to prevent lumping [10].

#### 1.4.2. LM Pectins

In LM pectins, less than 50% of the total carboxyl groups are esterified. They gel independently from sugar content and are chemically more stable to moisture and heat than are HM pectins [122]. They also are more resistant to pH than the abovementioned HM pectins, and gels can be obtained in a wide range of pH [10,123]. LM pectins can gel in the presence of divalent cations, usually calcium (Ca^2+^) and this gelling process can be easily reversed by adding monovalent ions like sodium (Na^+^) and potassium (K^+^) [124]. In these systems, gelation is due to the formation of intermolecular junction zones between pairs of carboxyl groups in the homogalacturonic smooth regions of different chains in close contact [34].

**Figure 2:**
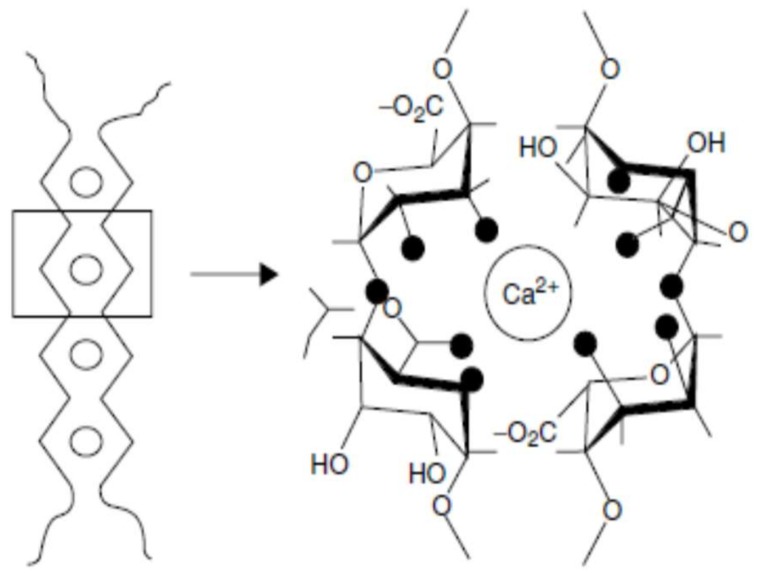
Structure of pectin of low methoxyl as call “the egg box” model. Adapted from [34].

The structure of such a junction zone is generally attributed to the so called ‘egg box’ binding process (Figure 2) [125,126]. Initial strong association of two polymers into a dimer is followed by the formation of weak interdimer aggregation, mainly governed by electrostatic interactions and ionic bonding of carboxyl groups [10]. The gel-forming ability of LM pectins increases with decreasing degree of methylation. LM pectins with a block wise distribution of free carboxyl groups are very sensitive to low calcium levels. Some plants like sugar beet and potato, present acetyl groups in their structure that prevent gel formation with calcium ions but give the pectin emulsion stabilizing properties [121]. The texture of low methoxyl pectin gels can be controlled by adjusting the calcium to pectin ratio. A high content of pectin with relatively little calcium will give an elastic gel, while the use of more calcium with a minimum of pectin will produce a much more brittle product, possibly with some syneresis [66]. Although sugar is not essential for gel formation in low methoxyl pectins, small amounts (10–20%) of sugar tends to decrease syneresis and adds desirable firmness of these gels, also when some sugar is present, the amount of calcium required to form gel is reduced. On the other hand, high concentrations of sugar interfere with gel formation because the dehydration of the sugar favors hydrogen bonding and decreases cross-linking by divalent ion forces [10].

#### 1.4.3. Ferulated Pectins Crosslinking

It is well known that ferulic acid is linked to polysaccharides like pectins in many plants and that it is possible to form gels through oxidative coupling [127,128,129]. These phenolic acids are very important components for the biology of the cell wall, as well as for its structure, because they can potentially cross-link polysaccharide chains through a dimerization reaction [102,105,130].

The cross-linking takes place through the formation of a covalent bond (mainly C–C) between two ferulate phenyl rings giving the formation of phenoxyradicals [131]. This oxidative coupling reaction is mediated by chemical or enzymatic oxidation and the gelation takes place at room temperature within a few minutes [82].

Gels are obtained for pectin concentrations approximately above 1% using a cross-linking agent such as polyphenol oxidase including laccase/O_2_, peroxidase/hydrogen peroxide or ammonium persulfate [105,128]. The oxidative coupling reactions of ferulated monomers lead to the formation of dehydrodimers isomers of ferulic acid. Variousauthors have reported four principal dehydrodimers in sugar beet pectins, such as 8-8′, 8-5′, 8-*O*-4′ and 5-5′ [112,129,130,131].

An adequate control of pectin concentration, amount of cross-linking agent and time of reaction are very important factors affecting the thickening and gel formation. These gels show interesting properties for food industry for its high water holding capacity [72], and thermostability feature, allowing the product to be heated while maintaining its gel structure [82]. Taking account of many sources reported of ferulated pectins, a third class of pectins should be considered as a function of gel formation.

## 2. Potential Applications of Pectin in Pharmaceutical and Biomedical Industry

Pectin, as a natural polymer in many fruits, possesses many interesting properties that have been widely exploited in food technology, and studied in the biomedical and pharmaceutical fields. The search for new and better ways to applications continue nowadays. Some of the major applications of pectin are summarized in Table 1.

### 2.1. Prebiotic Effect

In general, prebiotics are defined as indigestible food ingredients that beneficially affect health through various mechanisms [132]. One of them is their effect on colon microbiota populations. The consumption of pectins generates an increase in beneficial microbial populations in the gastrointestinal tract, increasing production levels of short chain fatty acids and gases such as methane, carbon dioxide and hydrogen, positively affecting health [133,134]. In addition to the above mentioned, pectins are considered as a soluble dietary fiber with several beneficial gastrointestinal physiological effects including the delay of gastrointestinal emptying, decreasing the time of gastrointestinal transit, reducing glucose absorption and increasing fecal mass [135,136,137].

Pectins have recently been classified as an emerging prebiotic and their potential in this area is being evaluated [138,139,140]. The main products derived from the intestinal fermentation of pectin as well as other types of dietary fiber are acetate, propionate and butyrate that a key role in the prevention and treatment of metabolic syndrome, intestinal disorders and cancer; a positive effect in the treatment of ulcerative colitis, Crohn’s disease, high blood pressure, diarrhea and obesity [141,142].

The beneficial effects of the pectin-derived oligosaccharides that work as prebiotics will depend on their chemical and physical characteristics, and be measured by key factors such as consumption kinetics, the distribution of metabolic products and the effects caused by bacterial populations [142]. Islamova et al. [143] compared the prebiotic activity of different pectin polysaccharides, concluding that the source of pectin have a significant effect on the prebiotic effect on different bacteria strains and all the pectin polysaccharides evaluated, compared to commercial prebiotics.

### 2.2. Effect on Glucose Levels

Several studies have shown the positive effect of pectin in the reduction of blood glucose. A study in diabetic rats revealed the hypoglycemic activity of low methoxyl and high molecular weight pectins isolated from the fruit of passion (*Passiflora edulis*); in addition, there were not affections in organs like kidney, liver and pancreas [144]. On counterpart, Makarova et al. [145], worked with healthy volunteers to evaluate the consumption of unripe apples powder, rich in pectin, and measured hypoglycemic activity. The results showed that the glucose metabolism was improved, increasing the excretion of glucose via urine. These authors suggest that this formulation can improve the health of patients with diabetes.

Wicker et al. [146] proposed that the hypoglycemic activity of pectins is mainly because they reduce the absorption of glucose in blood and therefore a decrease on insulin production by the pancreas occurs. In this regard, reduction of insulin levels also leads to the decrease on the activity of the HMG-CoA molecule that reduces the synthesis of cholesterol. On the other hand, Liu et al. [147] suggested that the mechanism might be regulated by the expression of PI3K/Akt signaling pathway in a study that showed the activity of citrus pectin decreasing the levels of glucose in blood and insulin resistance in diabetic rats after 4 week of administration.

In account of pectin effects on glucose blood levels, pectin composite materials have been proposed as delivery matrices for insulin. Maciel et al. [148] studied the delivery of hormone insulin encapsulated in nano- and microparticles of a chitosan-pectin complex, under simulated gastric conditions. The encapsulation efficiency was of 62% and small-sized particles between 240 nm and 1900 nm were obtained, releasing the drug over two h in controlled conditions. These authors proposed these polysaccharides system as a suitable candidate for the release of insulin under oral administration. In addition, Rascón-Chu et al. [149] proposed a pectin-arabinoxylans composite microbead for insulin carrying. The characteristics presented in the structure of the polymers rendered small and stable particle sizes without aggregation or coalescence for all of the treatment evaluated, but few with little size dispersion that may allow the controlled delivery of insulin consistently.

### 2.3. Effect on Cholesterol Levels

The study of the relationship between cholesterol and the consumption of pectins has been studied extensively over the years through studies in vivo and in vitro. Accumulation of cholesterol in the body is associated with the risk of cardiovascular diseases. A diet rich in soluble fiber such as pectin results in a decrease in total cholesterol and low-density lipoprotein (LDL) in the blood without affecting the high-density lipoprotein (HDL) [150]. In addition, pectin enhances the excretion of bile acids, a substance that helps to remove the excess of cholesterol on the body, lowering consequently the serum cholesterol levels [151].

Gunness and Gidley [150] have proposed three possible mechanisms in order to explain the effect of reduction of total cholesterol and low-density lipoprotein (LDL) by the consumption of pectin. The first is the inhibition of reabsorption of bile salts within the enterohepatic circulation. Second, the high viscosity of the pectin could in turn affect the glycemic response, by reducing the rate of glucose absorption. Finally, the selective fermentation of the fiber by the colonic microbial populations (prebiotic effect), produces short chain fatty acids, especially propionate which can inhibit the synthesis of cholesterol in the liver.

The properties of several types of pectins to reduce cholesterol are related first to their physicochemical properties, including viscosity, molecular weight and degree of esterification [146].

Pectins have the capacity to form viscous gels that bind cholesterol and bile acids, promoting their excretion and reducing reabsorption. The cholesterol absorption is further hindered by disruption of micelle formation, constituted by bile salts, phospholipids and fatty acids [152]. Both high methoxyl and low methoxyl pectins reduce total cholesterol, and they have no effect on high-density lipoproteins (HDL) which are beneficial to health, but diverse studies have shown that high methoxyl and high molecular weight pectins are more effective in reducing cholesterol than low methoxyl pectins [146].

**Figure 3:**
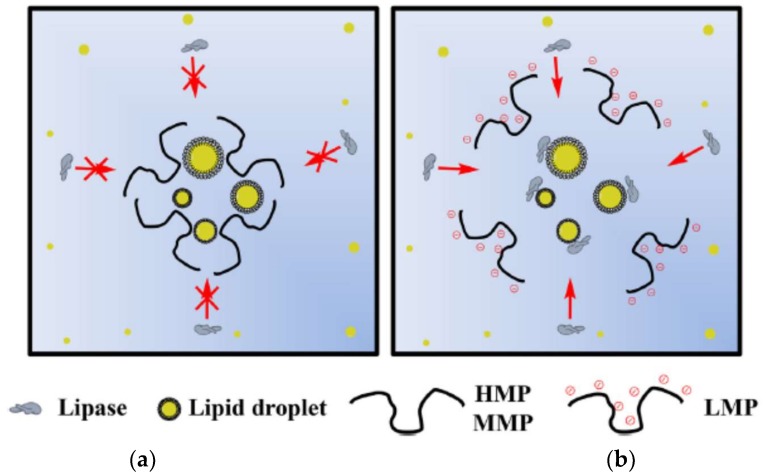
Inhibition o lipid droplet digestion by pectin isolated from banana passion fruit. (**a**) High and medium methoxyl pectins; (**b**) low methoxyl pectins [153].

Espinal-Ruiz et al. [153] analyzed different types of pectins from citrus and banana passion fruits and compared them under simulated gastrointestinal conditions measuring their impact on lipid digestion, and showed the nature of pectin affected the lipid digestion profile. The extent of lipid digestion was diminished with the increase of the molecular weight and pectin methoxylation. Pectins with higher degree of methoxylation affected the rheological properties of the gastrointestinal fluids, bringing an increase in the hydrophobicity of the molecule and decrease the number of negative groups. These authors suggest that having control of the lipid digestibility in the gastrointestinal tract can act as a functional food in the design of emulsion-based food and thereby promote health. In Figure 3, the authors shows the schematization of the action proposed for pectin in lipid digestion with HM, MM and LM pectins.

### 2.4. Removal of Metal Ions

Toxic metals as arsenic, cadmium, lead and mercury interrupt the normal functioning of the endocrine, neurological and immune systems, in addition to other functions in the human body. Normally heavy metal poisoning is treated by specific chelators such as ethylenediaminetetraacetic acid (EDTA), 2,3-dimercaptosuccinic acid (DMSA) orsodium 2,3-dimercapto-1-propanesulfonic acid (DMPS), which bind metals in the blood and facilitate their removal via urine and fecal routes. The levels of metals in the body can be reduced but these treatments may trigger secondary effects such as redistribution of metals in the brain or bones, reduction of essential minerals, disturbances in gastrointestinal function and skin rashes [154].

Pectin has metal- binding capacity, aids in the elimination of heavy metal ions, and is considered a reliable alternative to conventional chelators without secondary effects [155,156]. The ability of pectin to reduce absorption and the bioaccumulation of toxic metals is attributed to pectin binding the metals in the digestive tract and preventing their absorption while facilitating their elimination in the feces. More specifically, the basic mechanism proposed for the interaction of molecules of pectin and metal ions is the formation of the “egg box” structure;this mechanism assumes that four to six active residues of pectin units link a metal ion. The chemical structure, physicochemical properties and high heterogeneity of pectin in terms of molecular weight, degree of esterification, dispersion, and uronic acid content may hinder the metal-binding capacity of pectins [18]. Therefore, molecular tailoring and transformation is needed to render low methoxy pectin more effective [157].

Kyomugasho et al. [158] evaluated the effect of nanostructures of different pectins with low degree of esterification on mineral bioaccessibility in anin vitro model, showing that decrease in the degree of methylesterification of pectin results in a decrease on mineral bioaccessibility due interactions between pectins and ions. In addition, increments in the pectin concentration decreased the mineral-bioaccessibility in the digestive tract. The optimal in vitro bioaccessibility of mineral ions may be achieved controlling the structure of pectin.

**Figure 4:**
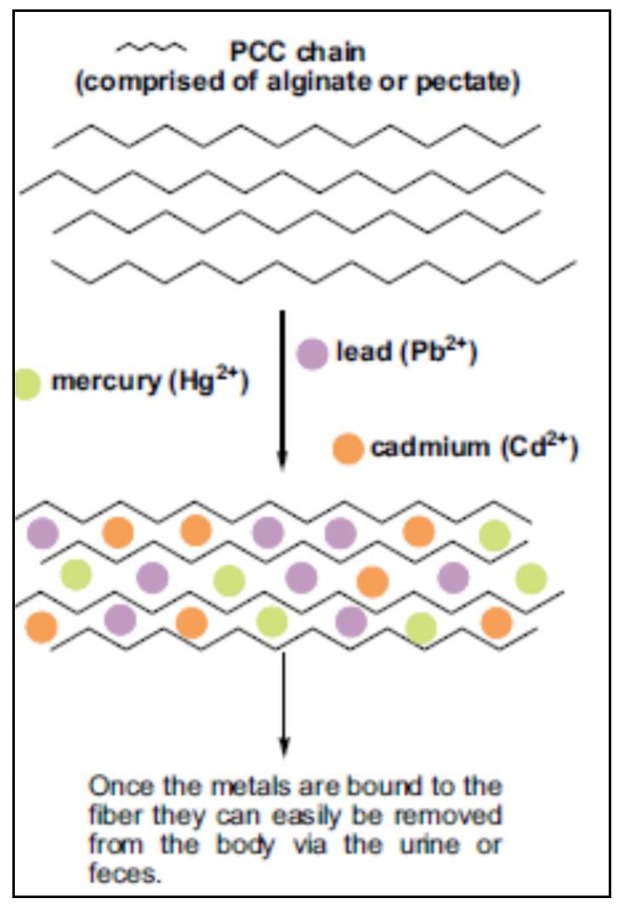
Entrapment of toxic metals in the call “egg box” structure formed by pectate or alginate. The positively charged metals are bound to the fiber chains and eliminated from the body [159].

The effects of modified pectins on the excretion of toxic metals in humans have been studied. Eliaz et al. [159] evaluated the oral administration of pectins with low degree of esterification (3.8%) and low molecular weight in humans with normal levels of metals, obtaining promising results increasing significantly the urinary excretion of toxic metal after six days of administration. These authors infer that the presence of rhamnoglactoturonan II rich in free carboxyl groups in pectin contribute to the chelation of metals. Figure 4 shows the proposed mechanism of action of fiber chains to entrap toxic metals as mercury, lead and cadmium.

For its part, in a study conducted by Zhao et al. [160], citrus pectin was administered orally to children hospitalized with toxic levels of lead in blood, revealing that all subjects studied had an increase in urinary lead excretion followed by a significant decrease in blood levels. In addition, no side effects were reported on negatively affected health. This study shows the effectiveness of modified citrus pectin as a chelator of lead, for its optimal structure, efficient for the chelation of heavy metals. The mechanism is still a subject of interest for science.

The presence of toxic metals like zinc in aqueous solutions derived from industrial waste, also represents a high risk to environmental and human health due to large excess of zinc, may be carcinogenic. The use of pectin with low degree of esterification (close to zero) with pH in a range of 4–7 can be very effective for elimination of heavy metals in aqueous disposals acting as sorbents [161].

### 2.5. Cancer Prevention

Another promising feature of pectin is its apparent synergism and chemoprotective action on metastasis of cancer and the growth of primary tumors in multiple types of cancer in human and animals. Leclere et al. proposed that pectin exerts synergism in combination with conventional anticancer drugs. These mechanisms are still under study and seem to depend on the structure of the pectin to yield various active fragments that can antagonize an active site or bind molecules, which can induce cellular apoptosis and inhibit tumor metastasis [162].

In the literature there exists a considerable number of reports that provide evidence for a role of pectin and modified pectinin the inhibition of different types of cancer. The list includes prostate cancer [163,164], colon cancer [165,166], pancreatic cancer [167], breast cancer [168] and metastasis [169]. In addition, it has been reported that this polysaccharide has the capacity to reduce cell proliferation, migration, adhesion, and induce apoptosis in a variety of cancer cell lines [13].

Pectins can be used in the form of dietary fiber and, since pectin is not digested in the gastrointestinal tract, it could protect cells from mutagenic phenomena in at least three ways. First, pectin is fermented in the colon by bacteria, and one of the products generated by this fermentation is butyrate, which inhibits colon inflammation and prevents carcinogenesis; then pectin and mainly modified pectins can interact with galectin-3 to inhibit cell metastasis; and, finally, the induction of apoptosis in cancer cells can be generated [162].

**Figure 5:**
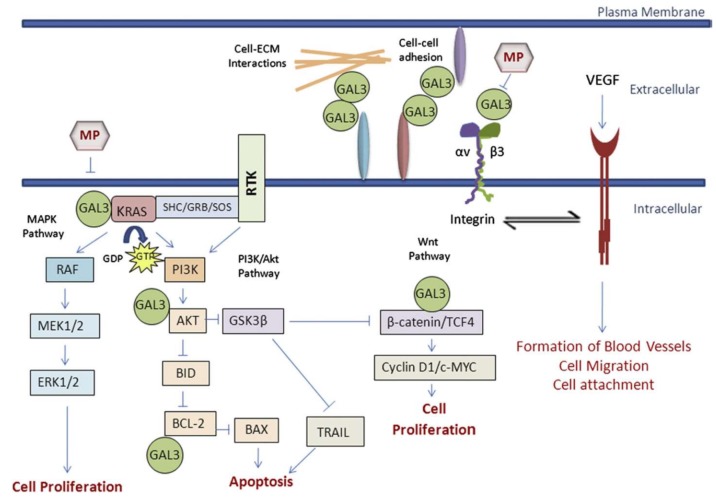
Roles of galectin-3 at extracellular and intracellular levels. At extracellular level Gal3 participates in cell adhesion. In intracellular level Gal3 activates different signalization pathways as MAPK, PI3K/Akt and Wnt that induces cell proliferations, apoptosis or bind to integrin receptors that activate VEGF to induce angiogenesis [198].

Modified pectin has shown to be more effective against cancer, when shorter, low branched, water soluble and galactose-rich modified pectin polysaccharide fractions are obtained by pH and temperature treatments. They have the ability to access and interact tightly with galectin-3 (Gal3) [170]. Galectin-3 is a type of lectin protein that is implicated in cancer, immune function and inflammation. High levels of galectin allow a greater adhesion and agglutination of cancer cells in the site of metastasis. Modified citrus pectin appears to act as a binding agent for the galectin-3 receptor site, binding it together and blocking the ability of cancer cells to bind to another site. This prevents the addition of tumor cells and their subsequent addition to endothelial cells [163]. Figure 5 shows the different pathways of action of galectin-3 at extra and intracellular levels.

The relationship between the structure ofmodified pectin and its inhibitory activity on galectin-3 was investigated in several studies [171,172]. In a model of liver metastases and colon cancer in mice, the level of expression of galectin-3 was higher compared to that of normal mice. In the study, modified pectin showed an effective inhibition of liver metastasis in a dose-dependence manner and also of the tumor volume of colon cancer in the model [169].

In this regard, Maxwell et al. [173] conducted a study to evaluate on the antiproliferative effect of different pectin extracts on colon cancer cells. The expression of genes involved in the cell adhesion was investigated. Briefly, the structure of pectin has an important role in the regulation of the antiproliferative activity. The RGI-region of pectin decreased the proliferation of carcinogenic cells; also, the expression of the gene ICAM1 decreased significantly in the colon cancer cells treated with RG-I, suggesting that this gene may play a role in the reduction in cell growth. These findings support the theory that HG segments in RGI, as well as the presence of neutral sugar side chains, are essential for pectin bioactivity.

These same authors evaluated in 2016 the antiproliferative activity of a pectin extract from sugar beet under alkali treatment in colon cancer cells. The alkali treatment decreased the degree of esterification of pectin, which increased the observed ratio of rhamnogalacturonan I (RGI) to homogalacturonan. Interestingly, the fractions had a significant increment on triggering apoptosis without any affection on normal cells’ cycle [174].

In studies conducted with humans, Guess et al. [163] evaluated the effect of modified citrus pectin (Pecta-Sol^®^ (Santa Rosa, CA, USA) in patients with prostate cancer showing that modified citrus pectin (MPC) can increase prostate-specific antigen (PSA) doubling time in 7 of 10 men (with biochemical recurrence of prostate cancer following local therapy) after taking MCP for 12 months, compared with before the take of MCP. This was the first published human clinical trial to use MCP to demonstrate it effect on the time of duplication of prostate specific antigen.

In a pilot clinical trial the use of modified citrus pectin in patients with advanced solid tumors was evaluated, in terms of tolerability, clinical benefit and antitumoral efficacy. The results obtained showed a significant improvement of quality of life and stabilization of patients. In addition, no patient developed any severe therapy-related adverse events. In particular, one patient in the latter study, who had advanced and hormonal resistant prostate cancer, had a 50% decrease in serum PSA level with significant improvement in his quality of life and decrease of pain after 16 weeks of treatment. Most of the patients had improvements in their quality of life [175].

### 2.6. Pectin as a Drug Controlled Release Matrix

The use of pectin as a matrix for the controlled delivery of drugs has raised interest in recent years mainly in biomedical applications such as drug delivery [14,18], gene delivery [176], tissue engineering [177,178], wound healing [179] and wound dressing material [180], for its capacity to form stable gels and its simple gelling mechanism.

For some pectin hydrogels applications to drug delivery, pectin with a high degree of esterification has been used, but variables such as high molecular weight and poor solubility in water, can considerably affect the encapsulateddrugs, leading to drug migration, early release or erosion of the cover. So in terms of desirable structure of pectin, the utilization of pectin with low degree of esterification is preferable, for its low molecular weight; also amidated pectins with low methoxy can influence in the junction zones [146,180].

Pectin has the ability to form gels in presence of calcium to create a water-insoluble cross-linked polymer called calcium pectinate (caP). This material is considered an attractive candidate for colonic drug delivery and forms hydrogel which can encapsulate drug molecules [16,181]. Penhasi et al. [182] studied the enzimatic degradation of calcium pectinate films through an ex vitro model simulating the colon’s environment, finding that the enzymatic degradation of the films was directly related with the concentration of sodium chloride in the solution. caP films with high concentration of sodium chloride were not degraded independently to the degree of methoxylation of the pectin, so it could be used for the release of water-soluble bioactive materials through the gastrointestinal tract. On the other hand, caP films with low concentration of sodium chloride presented high biodegradability being highly degraded by enzymes of the colonic bacteria and can be use as delivery system for hydrophobic active material.

As a natural polymer, pectin has interesting properties such as biocompatibility, mucoadhesiveness, safety, inertness, and has the ability to form gels in acid environments; hence, it is considered a suitable candidate for drug delivery models [18]. The encapsulation of bioactive compounds in biomaterial carriers as pectin has been studied. The De Souza [183] group worked with pectin microparticles elaborated by the electroaspersion technique with the aim of using them as a directed release medium where an active compound from mango was encapsulated, mangiferin, a compound of therapeutic interest for its antioxidant, prebiotic, antiviral, antitumor and anti-allergenic properties. For its part, the release of theophylline coated in pectin and calcium pectinate films were evaluated in an in vitro drug release study. The drug release was successful in an acidic medium (pH dependent) in a bi-layer film with combination of pectin and calcium pectinate [184].

Pectin has also been used as a targeted delivery vehicle for cancer; Jantrawut et al. [185] evaluated the rutin anticancer activity encapsulated in low methoxyl pectin beads on different cancer cell lines. The results obtained indicated, that rutin encapsulated in low methoxyl pectin showed a higher anticancer activity than the non-encapsulated rutin.

This polysaccharide has been studied and used for the specific delivery in nasal, ocular and oral treatments. The mucoadhesiveness of pectin gives it promising uses in nasal treatments in local diseases as allergies, nasal congestion and nasal infections [186], so this particular polymer has been proposed as a carrier to achieve a controlled release of active principles, through hydration/diffusion mechanisms of the mucosal tissue. The mucoadhesive potential of pectin with low degree of esterification may be due to associations by hydrogen bonding between pectin free acid groups and mucin in aqueous medium and thought adsorption mechanism of pectin on the mucin molecule [186]. Pectin has been proved as nasal spray to improve the analgesic onset, treatment efficacy and acceptability to treat cancer pain, where a rapid drug release is requested [187,188,189].

The use of pectin has been evaluated in ocular disorder treatments as a new strategy to enhance contact time and drug penetration in the eye, owing to the fact ophthalmic drugs present certain disadvantages likepoor bioavailability due to the protective mechanisms of the eye [17,190]. In a patent obtained by Ni and Yates [191], the formation of a pectin gel in situ was evaluated to apply it in liquid form and then gels in the eye. The presence of electrolytes and pH of the tear film conducted to the gelation of pectin. For its part, preliminary studies were assessed on pectin microspheres as an ocular delivery system for piroxicam. This system, can ensure high bioavailability, also has been shown to reduce some disadvantages of other ophthalmic delivery systems (pomades, inserts, liposomes) such as vision blurring, need for insertion and removal, and stability problems [192].

**Figure 6:**
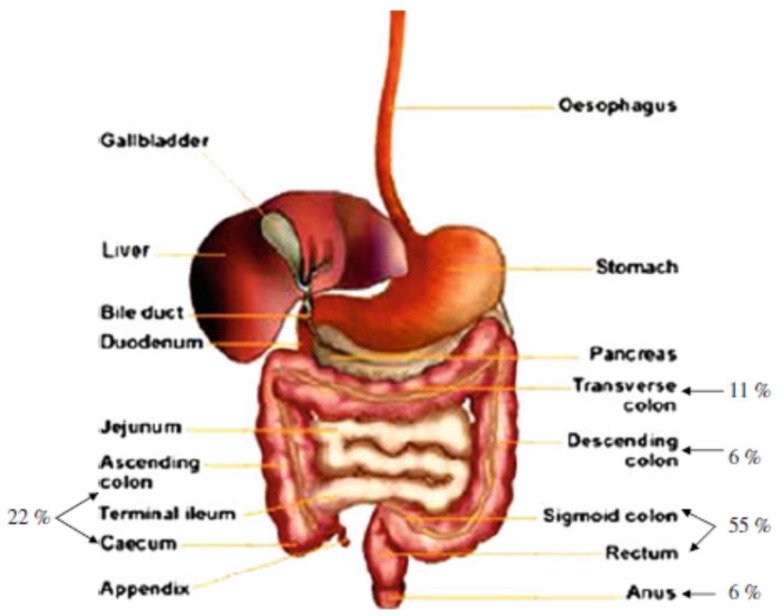
The gastrointestinal tract and its probable distribution in the percentage of colon cancer [195].

As mentioned above, pectin has the ability to traverse unaltered most of the digestive tract so it has acquired great relevance in the use as a matrix for controlled release of drugs directed to the colon, to treat diseases such as colon cancer (Figure 6), irritable bowel disease, Crohn’s disease, among others [68]. Diverse studies have been reported in the last decade in the use of pectin for drug release and/or treat colon cancer [180,193,194,195].

In a study performed by Dev et al. [196], the ability of pectin matrices coated with Eudragit S 100 to release 5-FU in the colon was evaluated. The results obtained showed that this formulation has a latency period of 4 h, enough time for the system to release the drug in the colonic zone. Likewise, the cytotoxicity studies carried out with a human colon cancer cell line (HT29) showed that the presence of pectin in the formulation significantly reduces the cytotoxicity concentration in cells CTC50%, which would enhance the activity of this formulation compared to a formulation without pectin.

In this regard, experiments carried out by Jung et al. [197] evaluated the entrapment of indomethacin in hydrogel beads of modified citrus pectin in an in vitro study, obtaining high entrapment efficiency. Also, the hydrogel beads were able to protect drug from the conditions of the simulated gastric environment, being a suitable candidate for a colon targeted drug delivery system. Nevertheless, pectin by itself is not totally effective in all cases to achieve formulations capable to reach the colon in unaltered. Some authors have reported matrix formulations of pectin coated with polymers that protect the pectin complex during its journey through the upper digestive tract. The interaction of pectin with some food polysaccharides is described in the text below.

## 3. Pectin Composites

The interaction between biopolymers has been widely studied for its great scientific importance and for its relevant new applications. For this, it is very important to understand the interactions that happen among different mixtures of polysaccharides, which, can result commercially attractive in the development of formulations with better stability or more desirable textures and above all, obtain a higher cost benefit, decreasing the use of expensive synthetic biopolymers by replacing them with cheaper and safer ones [199].

There are two essential types of interactions between polysaccharides: (1) attraction between the molecules, that occurs when the polymers are attracted to each other having an energetically favorable association; and (2) repulsion by steric exclusion, where the polymers repeal and exclude each other from the space they occupy [199].

The combination of different types of biopolymers is in constant growth, and diverse materials have been formed with different polysaccharides as starch, pectin, chitosan, cellulose, among others. Pectin is a biopolymer that has aroused greater interest in the formation of new composite material for its physicochemical and ionic characteristics [8]. Some interactions between pectins and other polysaccharides with potential uses in food and pharmaceutical industries are described below.

### 3.1. Pectin/Alginate

Pectin- alginate systems were the first reported mixed gelsand are widely studied to this day. Diverse authors have reported that the structural properties of this type of mixed gels depends largely on the pectin-to-alginate ratio, the degree of esterification of pectin and the mannuronic and guluronic acid proportions of alginate [200,201]. Toft [200] reported that mixtures of HM pectins and alginates with high content of l-guluronic acid gels sinergistically under certain conditions in which neither the pectin nor the alginate can form cohesive gels by their own.

HM pectins require high solids concentration and low pH for gelling, whereas gels obtained from mixtures of HM pectin-alginate are largely independent of the solids content and also less dependent on pH, representing an advantage [201]. Also, the presence of alginate enhances the structure development rates during gelation of pectin-alginate-sugar systems [202].

The strongest gels are formed when the mannuronic/guluronic acids ratio is low and pectin have high degree of esterification, and when these polysaccharides have similar levels, the optimum interactions occur [203]. It is important to mention that alginate with higher guluronic acid content, leads to the formation of gels with higher stability. Morris [204] evaluated the formation of a gel network with pectin with high degree of methoxylation and alginate with high guluronic acid (70% guluronate) obtaining gels two to three times stronger in terms of rigidity and break point, than those formed of alginate with high mannuronic acid (60% mannurate), under equal pH conditions.

In pectins with low degree of esterification, a much lower pH is needed to form gels with alginates. In these mixed gels, the melting point occurs when the pH decreases, and the gel structure can be kept at 100 °C under sufficiently acidic conditions [204].

One of a few determining factors to explain the synergistic interaction between the galacturonic acid chains of pectin and the guluronic acid blocks of alginate, is the structural similarity between these polymers, being both polyuronates. In fact, pectins and alginates can undergo intermolecular binding to rise coupled networks [203]. The interaction between pectin and alginate is enhanced as the proportion of these sequences is increased. The gelation mechanism between pectin and alginate involves intermolecular junction zones formed by the union of polyvalent cations (e.g., Ca^2+^) with guluronic acid blocks of alginate or galacturonic acid residues in pectin of adjacent polymer chain, forming an “egg box” configuration [205,206].

**Figure 7:**
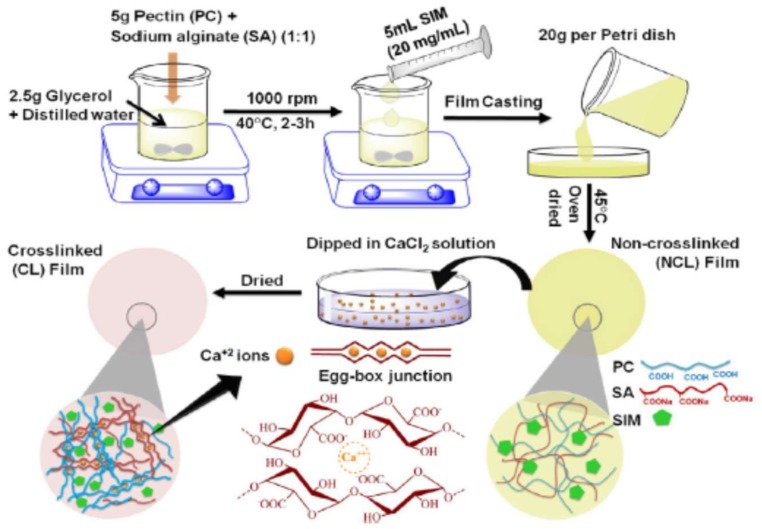
Diagram of pectin and sodium alginate film preparation and crosslinking process forming an egg-box junction between the Ca^2+^ ions [207].

Rezvanian et al. [207] present a schematization of the preparation of a pectin/alginate hydrogel forming the above mentioned egg box structure and is shown in Figure 7.

The mixture of pectin-alginate can lead the formation of thermoreversible gels making it one of the most interesting features for the food industry, including preparation of cold-setting fruit gels, stabilization of acidic emulsions like salad cream or mayonnaise. Also, these mixtures can be used in low sugar or calorie jams and jellies [208].

Other important uses of these polysaccharide mixtures are in the pharmaceutical industry as matrices for the encapsulation of diverse active ingredients [209,210] and as drug delivery systems [211], mainly as colon-specific drug delivery [212]. This is due to the fact that pectin and alginate can remain intact in the upper gastrointestinal tract and are degraded by specific enzymes produced by bacteria that inhabit the human colon [213]. Also, it has been proved that mixtures of citrus pectin/alginate have the ability to chelate heavy metals [214].

Many examples have been reported in this decade. Hsu et al. [215] developed and evaluated microspheres of pectinate/alginate coated with the polymer Eudragit S100 for the delivery of cisplatin in the colon, through the technique of electrospraying, obtaining microspheres with homogeneous sizes and pH independent under simulated gastric conditions. Furthermore, Belščak-Cvitanović et al. [216] evaluated the microencapsulation of polyphenols and β-carotene from *Taraxacumofficinale* L. in a binary mixture of pectin and alginate, achieving the entrapment of both active compounds. As the authors state, the binary mixture enabled the best (prolonged) release profile of these compounds in simulated gastrointestinal fluids. Galus and lenart [217] characterized diverse physical properties of composite edible films of pectin and alginate concluding that the combination of both polysaccharides gave continuous, homogenous and transparent films. Seixasa et al. [218], created biofilms composed of alginate and pectin obtaining a composite film homogeneous, improving its physical characteristics with potential applications in the food industry as covering of food, drug coating, among others. Many more potential applications are still being assayed for new food products and drug carrier design.

### 3.2. Pectin/Chitosan

**Figure 8:**
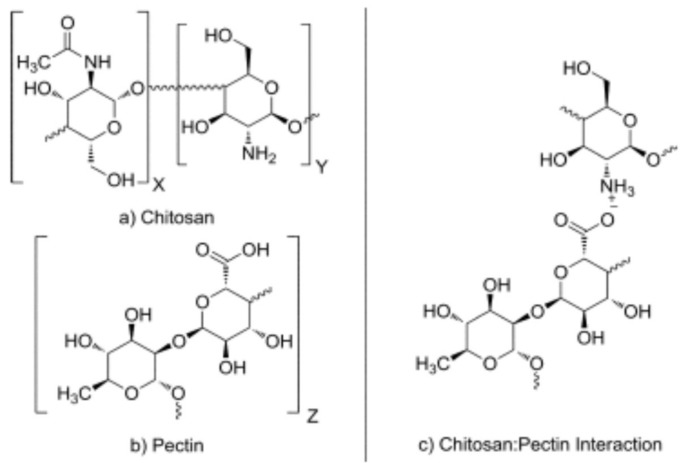
Chemical structure of (**a**) polycation chitosan and (**b**) RG-I region of polyanion pectin and (**c**) electrostatic interactions between pectin-chitosan [221].

Pectin and chitosan are polysaccharides which can form complexes useful for their biodegradability, biocompatibility and non-toxicity. Chemically, these biopolymers are considered as polyelectrolytes, so they have the ability to form the called “polyelectrolyte complexes” [219]. Chitosan is a weak polybase and pectin a weak polyacid, so in solution, this can lead to the formation of electrostatic attractions between the amino groups, charged positively (NH^+3^) in chitosan and the carboxyl groups charged negatively (-COO^−^) in pectin [220] (Figure 8).

Different interactions can occur between the groups in pectin-chitosan complexes:van der Waals, electrostatic, hydrophobic, hydrogen and coordination bonding. The presence of these polar functional groups results in a very strong intermolecular interaction and highly ordered orientation of the rigid chain polymers [222,223].

The stability ofthese pectin-chitosan complexes depends mainly of the pH, charge density, concentration of both polymers and on the ionic conditions of the medium. In addition, temperature, concentration of certain molecules, among other environmental conditions may play a significant role in this mixed gels stability [224,225,226].

There are extensive investigations dedicated to the studies of the physicochemical properties of the interaction between pectin and chitosan [222,227,228,229]. Rheological investigations have shown that the gelation is thermoreversible when the pH value is below two and that the gelling temperature is dependent on the weight ratio of the polysaccharides [230,231]. Marudova et al. [232] studied the interactions within gel formation of chitosan and pectin, concluding that chitosan can act as an effective crosslinker of pectin networks in acidic pH (5.6), and that the gelation behavior depends of the degree of esterification of pectin, showing a dependency on charge on the pectin.

These polyelectrolyte complexes have many uses in diverse fields like food industry [233], biomedicine [234,235], and pharmaceutical industry, especially as a drug delivery vehicle [231,236] and colon-specific drug delivery [237,238,239]; these drug carriers include hydrogel, films, tablets, pellets and beads. The interest on this system for controlled release of drugs lies in the fact that both polymers have the capacity to protect the drug from being released in the upper intestinal tract and deliver it in the colon [238].

Kowanolek [220] studied the interaction between a chitosan/pectin complex exposed to UV radiation acting as a potential sterilizing agent on such biomaterials. The complex showed higher resistance to UV action compared to polymers only; UV rays did not affect significantly the morphology and thermal stability of the complexes, which favors applications such as medical or pharmaceutical industry.

### 3.3. Pectin/Protein

The interactions between pectin and protein can occur through two different mechanisms in nature: associative phase and segregate phase separations, depending on the structural and ionic characteristics of the two components in the mixture. Associative phase occurs between two oppositely charged polymers (electrostatic interaction) and leads to the phase separation, where one phase is enriched with two different biopolymers. Associative interactions occur under conditions of pectin ionization (negatively charged) and for proteins/peptides carrying out positive charges (below their isoelectric point) [240,241]. Segregative phase happens in two ways, due to strong electrostatic repulsion (between two similarly charged biopolymers) or for very high steric exclusion (between two neutral biopolymers) [242].

Another type of interactions can occur between proteins and polysaccharides, such as covalent bonds, known as conjugated, forming very stable structures. Also non-covalent interactions such as hydrophobic, hydrogen bonding and van der Waals forces can occur [243].

Diverse physicochemical factors can affect the formation and stability of the complexes such as pH, ionic strength, radio of pectin to protein, pectin and protein charge, and molecular weight, thus the predominant interaction is associated with ionic bonds and the charge density is determinant in the formation of the complex.Parameters like decrease of the charge density of pectin by partial esterification of the carboxyl groups, increment of the ionic strength or lowering pH can reduce the ability to interaction between thesebiopolymers [244,245,246].

Diverse authors have studied the interaction between pectin and proteins. McClements [247] established that mixtures of protein and polysaccharides can be used to fabricate particles, nanoparticles, among others, with a variety of different compositions, structures and dimensions in dependence of the nature of the polymers involved and the assembly principle used. In example, in 2009, Jones et al. [248] evaluated the interaction between a globular protein named β-lactoglobulin and beet pectin by thermal treatment, obtaining polymeric particles in a size range of 100–300 nm; these particles were stables over a relatively wide range of pH values, depending of the polysaccharide concentration. These authors infer that the particles obtained may be useful for encapsulation and delivery of bioactive food compounds.

In this regard, Jones et al. [249] obtained polymeric nanoparticles spheroids in shape from the interaction between β-lactoglobulin and pectin, concluding that the size and stability of the biopolymer particles formed depend on the type of pectin used, in this case pectins with high degree of esterification; this authors concluded that these particles can be useful in food and other industries as encapsulation or as lipid droplet mimetics.

**Figure 9:**
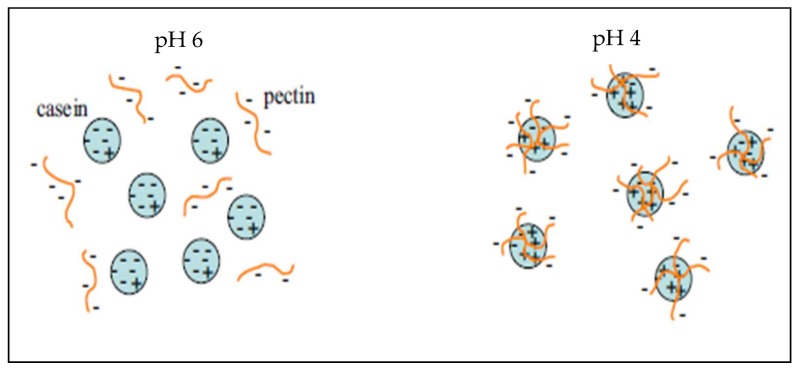
Stabilization of casein molecule with the pectin network. At pH 6.6 both polymers are negatively charged and repeal each other. At pH 4, pectin bound electrostatically to the positively charged areas of casein particles, preventing casein aggregates [252].

Microcapsules based on pectin/casein complex were evaluated for the release of the drug acetaminophen, obtaining spherical particles with a diameter of 5.18 μm after spray drying; said microcapsules can be used for the prolonged release of drugs throughout the gastrointestinal tract [250]. On the other hand, Jensen et al. [251] used pectin with high degree of esterification as a stabilizer of acidified milk, measuring the adsorption of the pectin onto casein aggregates, inferring that the adsorbed pectin formed a multilayer on the casein aggregates. Figure 9 shows the electrostatic interaction that occurs between the negatively charged pectin and the positively charged casein molecules at different pH values.

### 3.4. Pectin/Gelatin

**Figure 10:**
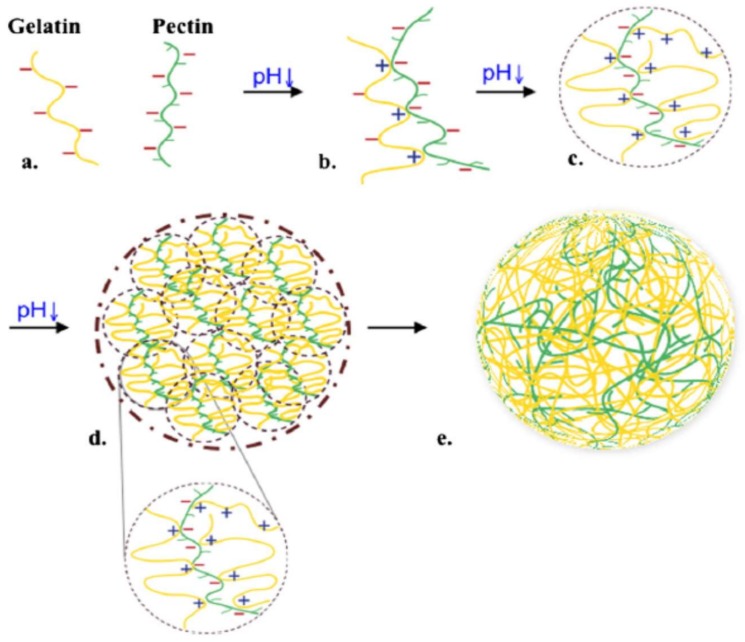
Schematic illustration of the electrostatic interactions that occur during acidification of gelatin and pectin mixtures (**a**) gelatin and pectin molecules exist as individual molecules in solution due to electrostatic repulsion; (**b**) gelatin–pectin soluble complexes are formed due to electrostatic attraction between positive charges on gelatin and negative charges on pectin; (**c**) soluble complexes merge and form gelatin–pectin complexes; (**d**) hydrogel particles form due to coalescence of sub-units; and (**e**) setting of internal structure as temperature cools down [256].

Gelatin is one of the food biopolymers which has been more studied. Chemically it is considered as a denatured collagen. The interaction between gelatin and pectin has been reported for several authors. These mixes of biopolymers are usually used in formulas of food products and the interactions between them are given through two phenomena to form a gel structure: the first phenomenon is the phase separation that includes two mechanisms type, the segregative (also called thermodynamic incompatibility) and the associative (also called complex coacervation) interactions (Figure 10). In an associative phase separation one of the separating phases is enriched in both polymers, while in a segregative phase each phase is enriched in one of the polymers [253]. Gilsenan et al. [254] studied these two interactions (associative and segregative) in LM pectin and gelatin, with the absence of calcium.

The second phenomenon is the gelation of each polymer or of the association of polymers together, depending of the type of interactions that occurs between them. The gelation process happens when the viscosity of the mixture increases followed by the formation of a network of infinite size, expanding the whole volume and finally stopping the phase separation phenomenon. Factors as rigidity, charge density, solubility may affect the way the polymers interact in the complex system [255].

Lui et al. [257] evaluated the rheological properties, microstructure and texture of different mixtures of gelatin and pectin concluding that the interaction between them in terms of cohesiveness and brittleness is on dependence of the radio of pectin to gelatin, increasing the hardness of the gels with the addition of pectin to the solution resulting in a synergistic effect, derived of the electrostatic interaction that might lead to the reinforcement of the gelatin/pectin network. For its part, diverse authors establish that the rheological properties of hydrogel particles are affected by parameters as particle shape, size and size distribution [256,258,259].

Gupta et al. [260] evaluated the cross linking reaction for the formation of a hydrogel of gelatin and pectin concluding that factors as reaction time, reaction temperature, pH of reaction and composition affect directly the interaction between these polymers. The fabricated hydrogel did not exhibit any phase separation, having promising uses in biomedical technology.

As mentioned above the gelatin pectin complex can be used in the food industry, for example to confer texture to food since these complexes have similar rheological properties as starch granules, a polymer widely used in the food industry [261,262]; or may be a promising product in the subject of healthy reduced-calorie foods for the source of obtainment, protein and dietary fibers [263]. Also, the gelatin/pectin interactions have been focus of investigations in the matters of pharmaceutics, as composites for controlled drug delivery [264] or microencapsulation of bioactive agents as lycopene [265], and in biomedicine for bone regeneration [266], or composites for wound healing [267,268,269].

### 3.5. Pectin/Starch

With respect to the pectin/starch systems, the compatibility or incompatibility among the two components is influenced by several factors, such as molecular structure of starch, degree of esterification of the pectin, the concentration of both polysaccharides, presence of other co-solutes (i.e., calcium or sugars), and the ionic characteristics of the media [199].

The rheological, physical and chemical properties of these mixtures have been widely studied; Autio et al. [270] evaluated the rheological, microstructural and sensorial profile of a mixture of starch and commercial pectin with low degree of esterification. Higher content of calcium (Ca) and the presence co solutes (sucrose) in the mixture had faster gelling rates than the gels formed separately. Gels with the lowest Ca content in the mixture affected the sensory profile obtaining gels smoother, more elastic and firm with respect to the other mixtures. Khondkara et al. [271] made a rheological characterization in terms of viscoelasticity of blends of starch and pectin, using sodium trimetaphosphate as cross linking agent. The use of a cross linker had a considerable influence on the rheological and physicochemical properties of the gels and the study revealed that the cross links between these polysaccharides can occur in mixed polymer systems.

A similar study was reported by Carbinatto et al. [272] who evaluated different mixes of pectin and starch with different degrees of cross linking (sodium trimetaphosphate). The results obtained by these authors coincide with the results obtained by the authors mentioned above; the use of sodium trimetaphosphate leads the formation of gels with higher thermal stability through the formation of covalent bonds.

Regarding the pharmaceutical uses of starch/pectin blends, the use of these polymers for the delivery of controlled drugs has also been studied [273]. Likewise, potential industrial uses of these polysaccharide combinations are include, as water soluble pouches for detergents and insecticides, flushable liners and bags, and in food industry as edible bags for soup and noodle ingredients [274]. Dafe et al. [275] lead an investigation on the encapsulation of probiotics (*Lactobacillus plantarum*) in a pectin/starch hydrogel by an extrusion method. The entrapment of probiotics in the matrix was successful and resisted the simulated gastric conditions, increasing the tolerance of *L. plantarum* to the strong acidic media.

On counterpart, Liu developed a composite material in 2014 for the delivery of ascorbic acid using a starch/pectin mixture, through the spray drying technique. The loading efficiencies and release profiles were dependent on starch/pectin ratio; also the starch/pectin radio had an impact on the size distribution of the microparticles [276]. Diverse authors have also studied the release of the drug diclofenac in matrixes of starch and pectin [277,278].

### 3.6. Pectin/Arabinoxylan

The existing information related to the interactions between pectin and arabinoxylan is low. To date it is known that the gelling mechanisms of both polymers, in part lies in the presence of ferulic acid in their structures which allows the formation of gels stronger and stable at temperature and pH conditions, mainly for arabinoxylans. It is well known that the main gelling methods for pectins will depend to a large extent on their degree of methoxylation, although as mentioned previously the presence of ferulic acid in some type of pectins gives it complementary mechanism of gelation by oxidative coupling.

**Figure 11:**
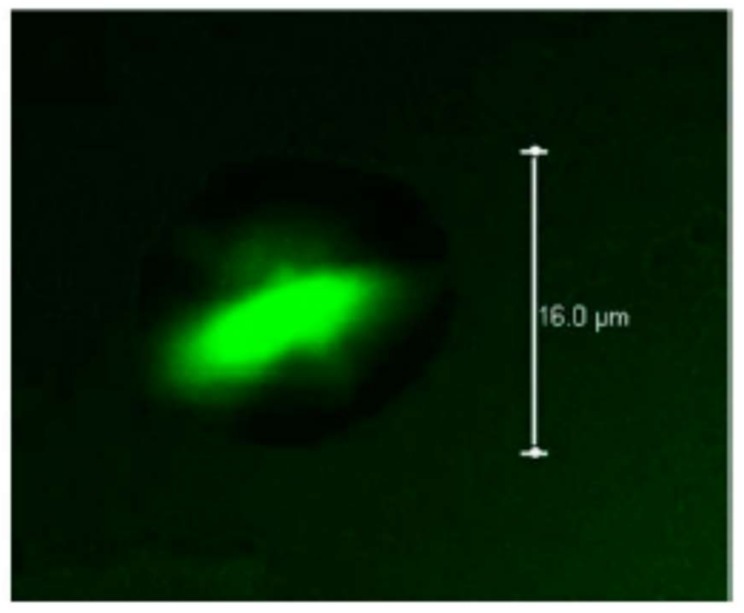
Confocal Laser Scanning Microscopy image. Protein insulin autofluorescence showed in the center of pectin/arabinoxylan bead (intense color) [149].

Up to this review, authors found little information on the interaction of these polysaccharides in relation to the covalent linkages that may occur between ferulic acid residues in theory. Nevertheless, some studies have been carried out for the evaluation of microspheres composed of pectin and arabinoxylans. For instance, Rascón-Chu et al. [149] evaluated the optimization of manufacturing parameters for core-shell type microspheres based on pectin/arabinoxylans, for encapsulation of insulin by coaxial electrospray method (Figure 11).

The authors suggested that the composite material formed is a suitable candidate for fabrication of microparticles with little variation in size, a critical feature for the prediction of delivery and accurate doses administration. Also, they envisioned further investigations in vitro and in vivo, to fully understand the diffusional kinetics and mechanisms of such a composite matrix.

## 4. Conclusions

Pectins are multifunctional polysaccharides widely used in food industry as an emulsifier, gelling agent, stabilizer, and/or thickener. In addition, due to their diverse chemical structure they can interact with equally varied type of molecules with promising uses in the pharmaceutical industry, health care and treatment, controlled drug delivery matrix of bioactive materials, and specifically colon targeted ones for their capacity to resist acidic conditions. The impact of this polymer is derived from its good gelation, low cost, non-toxicity, high stability, and biocompatibility. The extensive bioavailability in nature is remarkable, since pectins can be extracted almost from any source of dicotyledonous plants, and also from the transformation of agro-industrial wastes, such as husks and fruit bagasse and pomace.

Traditionally, pectins are obtained from citrus or apple fruits, but in recent years the use of unconventional sources has become an attractive form to obtain pectins from sunflower head residues, mango waste, amaranth, sugar beet, among others. The structural properties of pectin such as degree on esterification, molecular weight, pectin concentration, ion concentration and extrinsic factors as pH, ionic strength and temperature directly affect the gelation process. The presence of ferulic acid in the structure of pectin in plants like sugar beet gives them another gelling mechanism by the ability to gel by oxidative coupling.

The continuous study of pectin structure and its properties has enabled a mayor understanding of the complex heterogeneity of this polysaccharide, and continued development of new applications of pectin beyond the traditional uses in food, as pharmaceutical industries are increasingly embracing renewable and biocompatible materials. The perspectives of the new composites are the most promising in controlled and targeted delivery of therapeutic molecules with high potential in the reduction of doses of antibiotics required as well as new vehicles for oral administration of otherwise sensitive molecules like insulin. The anticancer, antiobesity and heavy metal-binding capacity has been well documented and will be of great impact in human health in the short term.

## Figures and Tables

**Table 1:** Biomedical/pharmaceutical properties and chemical features as degree of esterificacation of pectin extracted from diverse sources.

Biomedical/Pharmaceutical Applications	Pectin Source Obtention	Degree of Esterification (%)	Reference
Prebiotic effect	Orange peel wastes	NA	[138].
	Sugar beet pulp/lemon peel	NA	[139].
	Sugar beet pulp	NA	[140].
	Roots of *Bupleurum falcatum*	NS	[141,142].
	Apple pomace, tangerine peel and wild plant	NA	[143].
Glucose levels	*Passiflora glandulosa*	35	[144].
	Unripe apple	NS	[145].
	Citrus pectin	NS	[146,147].
Insuline delivery	Pectin/Chitosan	NS	[148].
	Pectin/Arabinoxylans	NS	[149].
Cholesterol leves	Soluble dietary fiber	NA	[150,151,152].
	Banana passion fruit	52	[153].
Metal removal	Seagrass, citrus pectin	60	[154,155,156].
	Seagrass *P. iwatensis*	2.54–6.91	[157].
	Commercial citrus/sugar beet	16,35	[158].
	Citrus pectin	3.8	[159].
	Citrus pectin	NS	[160].
	Citrus pectin	1	[161].
Cancer prevention	Modified pectin	NS	[162].
Prostate cancer	Modified citrus pectin	NS	[163].
	Citrus pectin (P-9135)	6.7	[164].
Colon cancer	Citrus pectin/Modified apple	30,60,90	[165,166].
Pancreatic cancer	Flowers of *L. japonica*	NS	[167].
Breast cancer	Pectic polysaccharides	NS	[168].
Metastasis	Modified citrus pectin	NS	[169,170].
	Modified citrus pectin	6.7	[171].
	Pumpkin	5.4	[172].
Antiproliferative effect	Citrus pectin	NS	[173].
	Modified sugar beet	18,55,57,62	[174].
	Modified citrus pectin	NS	[175].
Gen delivery	Amidated citrus pectin	26	[176].
Tissue engineering	Modified pectin with oligopeptides	LM	[177,178].
Wound healing	Amidated pectin	LM	[179,180].
Drug encapsulation	Citrus, pumpkin	48,44;8.1;30	[181,182,183,184,185].
	Unipectine OF300C		
Nasal drug delivery	Commercial pectin	29,38,70	[186].
	Fentanyl Pectin	NA	[187,188,189].
Ocular drug delivery	Mucoadhesive polymers	NA	[190,191].
	Cesapectin^®^	32	[192].
Colon drug delivery	Citrus pectin	36,25; 10,35	[181,193,194,195,196,197].

* NA: Not applicable; NS: Not shown; LM: Low methoxyl.
